# High-Quality and Intensive Post-cardiac Arrest Care Leads to Positive Outcomes: An Interesting Case Report

**DOI:** 10.7759/cureus.93428

**Published:** 2025-09-28

**Authors:** Maimouna Dhou Nourein, Weaam Ibraheem, Abdalla Diyab

**Affiliations:** 1 Rehabilitation Medicine, Salma Rehabilitation Hospital, Abu Dhabi, ARE; 2 Rehabilitation Medicine, Dumfries and Galloway Royal Infirmary Hospital, Dumfries, GBR; 3 Medical Research, California Institute of Behavioral Neurosciences and Psychology, Fairfield, USA

**Keywords:** critical care outcome, hypoxic brain injury, out-of-hospital cardiac arrest, post-rosc care, rehabilitation

## Abstract

Patients who experience out-of-hospital cardiac arrest have a low survival rate of around 10%. Ongoing research aims to prevent such events, ensure prompt responses, and improve outcomes when they occur.

We present a case of a prolonged out-of-hospital cardiac arrest with a downtime of 45 minutes that resulted in a poor EEG report and predicted a poor outcome; however, the patient has demonstrated good functional and physical recovery, which enables him to have a better quality of life. This case highlights the importance of prompt intervention in the early stages following cardiac arrest, as well as the necessity of an early holistic rehabilitation plan to maximize recovery and enhance quality of life. This approach can lead to positive outcomes despite poor initial objective tests.

## Introduction

The prognosis of post-cardiac arrest, either out of hospital or in hospital, varies according to the circumstances and causes of the cardiac arrest, the measures to prevent cardiac arrest and to improve the outcome if it has happened are among the important subjects to address in medicine. By examining the previous literature in PubMed for the last 20 years, we found that various factors affect the long-term prognosis of post-cardiac arrest, such as early identification of the cardiac arrest, prompt intensive care, and a holistic rehabilitation program following the event [1]. Those factors have been extensively studied earlier to improve the survival rates and quality of life.

Educating bystanders about the importance of early high-quality cardiopulmonary resuscitation (CPR) if they witness a cardiac arrest outside the hospital environment has been emphasized in a Danish study [2]. It was found that bystanders’ knowledge of CPR and intervention in the early minutes of cardiac arrest were significantly associated with increased survival following out-of-hospital cardiac arrest [2,3]. We also found that the implementation of a standardized post-cardiac resuscitation protocol is associated with better long-term prognosis [4].

This case report demonstrates how these factors affect the overall prognosis and quality of life for a patient who had a prolonged cardiac arrest outside of the hospital and had poor early neurological indicators for his recovery.

## Case presentation

**Figure 1:**
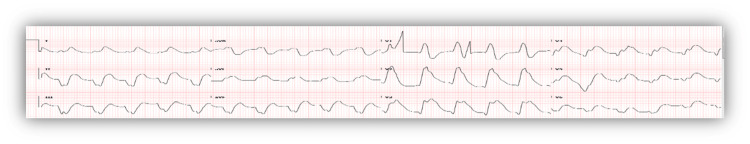
Sinus tachycardia, ventricular premature complex, IVCD, atypical RBBB baseline wander in lead V1, and ST elevation: considerations for an anterior injury IVCD: intraventricular conduction delay; RBBB: right bundle branch block

A 25-year-old male, previously healthy but a heavy smoker, experienced an unwitnessed cardiac arrest at his workplace. Upon arrival at the hospital, he was in asystole and had achieved return of spontaneous circulation (ROSC) after approximately 45 minutes. After achieving ROSC, he was found to have ST-segment elevation, indicating an anterior injury on electrocardiogram (ECG) (Figure 1).

**Figure 2:**
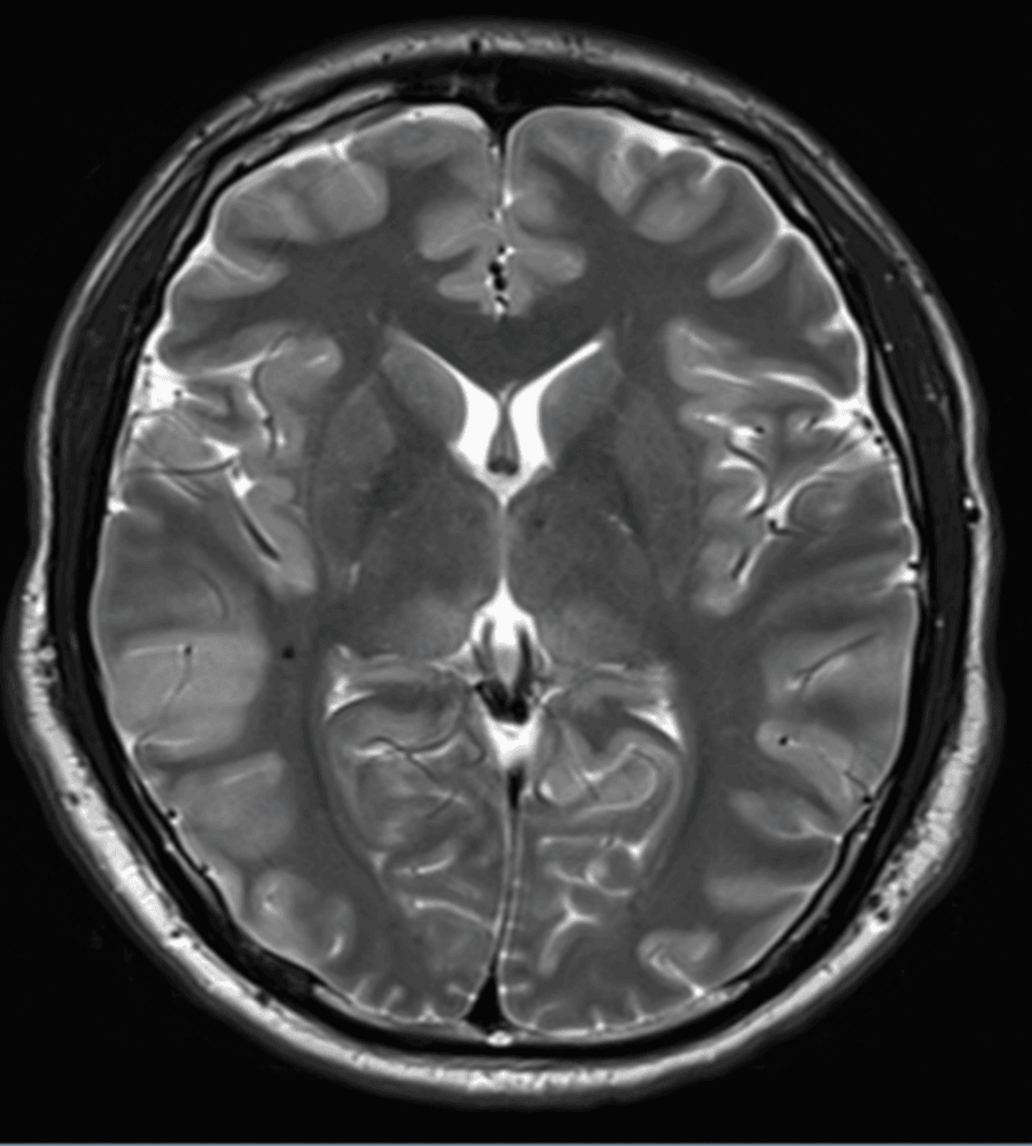
MRI brain; diffuse cytotoxic edema secondary to diffuse anoxic brain injury

The patient was directly moved to the catheterization lab and underwent early angiography, which showed a complete 100% thrombotic occlusion of the proximal left ascending artery. Angioplasty was carried out with a drug-eluting stent. This was followed by an echocardiogram, which showed significant left ventricular dysfunction. Post-procedure, the patient had oliguric acute kidney injury (AKI) with lactic acidosis, so he was started on continuous renal replacement therapy (CRRT). He also developed multiple episodes of generalized seizures, which were managed with anti-epileptic medication and sedation. Electroencephalogram (EEG) showed diffuse severe encephalopathy with a non-reactive background, and magnetic resonance imaging (MRI) showed gyral/cortical swelling/edema and a diffuse pattern of diffusion restriction involving the gray matter of almost the entire cerebral cortex in keeping with cytotoxic edema secondary to a diffuse anoxic brain injury (Figure 2).

He remained stable but with a low Glasgow coma scale of 9/15 for a few days. He required a tracheostomy, and then the ICU team decided to shift the patient to a long-term care (LTC) hospital for continuity of medical care and rehabilitation. Two weeks after he moved into the LTC hospital, he experienced an episode of instability during a hemodialysis (HD) session, characterized by tachycardia, hypotension, and oxygen desaturation. The HD session was immediately aborted; however, his oxygen saturation continued to drop, and his condition deteriorated rapidly, with tachycardia and low blood pressure, ending with ventricular fibrillation (VF) and cardiac arrest. CPR was started, and ROSC was achieved after one cycle. He was moved back to critical care and seen by the cardiology team, who advised continuing on conservative non-invasive management, as the VF was likely due to a cardiac scar rather than an underlying true ischemia, though there was no electrophysiological study or cardiac MRI done to confirm it. This time, the patient's condition was complicated by multiorgan failure, and he was expected to have a poor prognosis, especially in the context of anoxic brain injury.

Over time, he started to improve gradually. However, he required a percutaneous endoscopic gastrostomy tube for feeding and was later transferred back to the long-term care facility for continuity of care. During his stay at the LTC hospital, he was managed by a multidisciplinary rehabilitation team. Throughout this period, he demonstrated great improvement and achieved outstanding neurological outcomes. He was successfully weaned off the ventilator and decannulated. His physical function has improved with intensive physiotherapy and occupational therapy. Although he remains with severe cognitive dysfunction, his overall recovery was remarkable.

Upon discharge, he was able to walk unaided and feed himself orally. He needed assistance with activities of daily living, mainly due to his cognitive problems, although he could understand and obey commands and participated in social activities.

## Discussion

This patient had sustained an out-of-hospital cardiac arrest due to severe acute myocardial infarction, which lasted around 45 minutes, and he had another brief cardiac arrest two weeks later, when he revived after one cycle of CPR. The patient has had a complicated post-cardiac arrest course with cardiogenic shock, which led to multiorgan failure (liver shock, AKI requiring hemodialysis, and respiratory failure for which he was tracheostomized and kept on a mechanical ventilator).

The expected neurological outcome for this patient was poor, as suggested by the long-duration cardiac arrest, MRI, and EEG report that showed diffuse severe encephalopathy with a non-reactive background. Surprisingly, the patient showed an excellent response to the rehabilitation program and positive outcomes in quality of life and activities of daily living.

Almost one month after his first cardiac arrest, the patient was transferred to the rehabilitation ward, bedridden with a low level of consciousness, and on oxygen therapy via tracheostomy, and his feeding was through a percutaneous endoscopic gastronomy (PEG) tube.

In the rehabilitation ward, he was assessed for his rehabilitation needs and potential, and a detailed care plan was set by a multidisciplinary team, including physicians, rehabilitation nurses, physiotherapists (PTs), occupational therapists (OTs), speech language pathologists (SLPs), dieticians, and the psychosocial support team; the presence of the patient’s family was strongly encouraged to support him in the rehabilitation journey and to enable him to engage well with the activities. There were regular multidisciplinary team goal-setting meetings to ensure that the set goals had been achieved. During his stay in the rehabilitation ward, his medical condition and nutritional status were optimized as well.

Over time, he started to gain muscle strength to sit, stand, and walk, and with speech and language therapy, he was able to swallow and pass the video fluoroscopy study and start oral feeding.

Later, his tracheostomy was successfully removed and decannulated, and the PEG tube was removed as well. Although he remained cognitively impaired, one of the factors that helped the patient to engage more with rehabilitation is that he was cared for by a team that spoke the same language as him, which made it easy for him to understand and follow commands and be more motivated and engaged with the therapy. This highlights the importance of psychosocial factors in his rehabilitation journey. The rehabilitation team supported him in recovering and expressing his wishes and feelings. With his family's support, he was offered education about his condition and how to prevent such events in the future, such as smoking cessation and the importance of taking his cardiac medications and having a regular cardiology follow-up.

It is worth mentioning that the patient remained in the rehabilitation hospital for a total duration of one year, during which he participated in a comprehensive, multidisciplinary rehabilitation program. During his stay, there were no skin issues or spasticity/contractures that required treatment. He received intensive physical therapy three times per week, with each session lasting approximately one hour. Additionally, he underwent alternating weekly sessions of speech therapy and occupational therapy, tailored to his functional and cognitive needs. Throughout the rehabilitation period, the patient was under the regular care of the psychiatry team, whose involvement played a pivotal role in his neuropsychiatric stabilization and overall progress. He engaged in individual therapy and group support sessions. From a pharmacological standpoint, he was managed with antidepressants and mood stabilizers, namely, olanzapine and quetiapine. Quetiapine was discontinued three months prior to discharge, while olanzapine was continued as part of his ongoing psychiatric regimen.

To systematically monitor the patient's progress, a variety of validated scoring systems and outcome measures were utilized throughout his inpatient stay. The key metrics that highlight his functional improvements are as follows.

Barthel index: At admission, the patient scored 0, indicating complete dependence and a fully bedridden status. By discharge, his score had improved to 5, reflecting partial independence in select activities of daily living (ADLs) and minimal assistance required in others [5].

Elderly mobility scale (EMS): This tool, designed to assess mobility in frail older adults, yielded a discharge score of 14/20, signifying functional independence in basic mobility-related activities [6].

University of Kansas balance scale: At the time of discharge, the patient demonstrated a +4 in sitting balance and a 4 in standing balance, indicating marked improvement in both static and dynamic postural control.

Cognitive language assessment: At the time of discharge, the patient still presented with severe cognitive impairment. On formal assessment, his responses were largely non-purposeful, fragmented, or delayed. He demonstrated intermittent responsiveness to simple commands and to discomfort, with some responses being inappropriate. Nevertheless, he was able to recognize familiar individuals and engage with familiar routines, particularly in structured contexts. He maintained a cooperative demeanor throughout the rehabilitation process. With regard to cognitive-functional activity, he exhibited basic recognition of bodily needs and was capable of occasionally initiating simple actions. However, he remained highly dependent on assistance for most daily cognitive tasks.

Swallowing function (dysphagia): At discharge, the patient was diagnosed with mild oropharyngeal dysphagia, manifested by incoordination during the oral and pharyngeal phases of swallowing, without clinical evidence of aspiration. Functionally, he was able to consume a regular diet, reflecting sufficient compensatory strategies and safe swallowing function.

The patient was eventually discharged with good health status and an acceptable functional level that enabled him to carry out the basic activities of daily living with assistance, was able to feed himself orally, and, physically, he was able to transfer himself and walk independently, as well as participate in social activities and have a good quality of life.

Following discharge, the patient traveled back to his home country, with a recommendation to continue his physical/cognitive/cardiac rehab as an outpatient. Hence, we could not follow up with him down the line to see his further progress.

In summary, our case demonstrated a good outcome, and we can mention the factors that led to this result, such as early high-quality CPR and prompt post-cardiac arrest intensive care, which helped the patient survive the complicated course of his illness and be prepared for the rehabilitation stage. The early introduction of intensive rehabilitation also fostered good recovery. The patient was 25 years old, and according to previous research, patients aged less than 40 are associated with a better long-term prognosis post-cardiac arrest [7]. His young age enabled him to respond well to the rehabilitation plan and recover. Besides the early percutaneous coronary intervention (PCI) that he underwent post-cardiac arrest, which had a significant aspect as well [8], the presence of psychosocial support from the family and the team had clearly enhanced his engagement with the rehabilitation plan and, in turn, maximized his recovery.

Interestingly, the factors that can affect the prognosis of cardiac arrest can be categorized as (A) pre-cardiac arrest, such as age, cause of cardiac arrest, and other co-morbidities; (B) during the cardiac arrest, such as early, high-quality CPR; and (C) post-cardiac arrest, including early PCI/treatment, post-resuscitation care, and rehabilitation programs.

## Conclusions

The survival rate and outcomes following a prolonged cardiac arrest are generally poor, and not all patients experience the same recovery. In this article, we report a case of a young patient who was a heavy smoker but managed to survive after a prolonged cardiac arrest. Despite his EEG findings indicating severe hypoxic encephalopathy and poor outcomes, he surprisingly demonstrated good outcomes. Several factors contributed to his positive neurological recovery, including his young age, early coronary angiography, prompt neuro-cardiac-muscular rehabilitation, strong family support, and the collaborative efforts of the multidisciplinary team.

This case demonstrates that favorable outcomes following cardiac arrest can be maximized through effective teamwork and coordinated efforts from the moment of the cardiac arrest until the patient is discharged from the rehabilitation ward. Multidisiplinary rehabilitation can help improve independence and quality of life despite initial poor neurological indicators, such as the EEG, in this case.
